# Germinated and Ungerminated Seeds Extract from Two* Lupinus* Species: Biological Compounds Characterization and In Vitro and In Vivo Evaluations

**DOI:** 10.1155/2016/7638542

**Published:** 2016-12-20

**Authors:** Bogdan Andor, Corina Danciu, Ersilia Alexa, Istvan Zupko, Elena Hogea, Andreea Cioca, Dorina Coricovac, Iulia Pinzaru, Jenel Marian Pătrașcu, Marius Mioc, Romeo Teodor Cristina, Codruta Soica, Cristina Dehelean

**Affiliations:** ^1^Department of Orthopedics, University of Medicine and Pharmacy “Victor Babeş”, Eftimie Murgu Square, No. 2, 300041 Timişoara, Romania; ^2^Department of Pharmacognosy, University of Medicine and Pharmacy “Victor Babeş”, Eftimie Murgu Square, No. 2, 300041 Timişoara, Romania; ^3^Department of Food Control, Banat's University of Agricultural Sciences and Veterinary Medicine “King Michael I of Romania” from Timisoara, Calea Aradului No. 119, 300641 Timisoara, Romania; ^4^Department of Pharmacodynamics and Biopharmacy, University of Szeged, Eötvös u. 6, Szeged 6720, Hungary; ^5^Department of Microbiology-Virology, University of Medicine and Pharmacy “Victor Babeş”, Eftimie Murgu Square, No. 2, 300041 Timişoara, Romania; ^6^Department of Pathology, “Iuliu Hatieganu” University of Medicine and Pharmacy, 400006 Cluj-Napoca, Romania; ^7^Department of Toxicology, University of Medicine and Pharmacy “Victor Babeş”, Eftimie Murgu Square, No. 2, 300041 Timişoara, Romania; ^8^Department of Pharmaceutical Chemistry, University of Medicine and Pharmacy “Victor Babeş”, Eftimie Murgu Square, No. 2, 300041 Timişoara, Romania; ^9^Department of Pharmacology and Pharmacy, Banat's University of Agricultural Sciences and Veterinary Medicine “King Michael I of Romania” from Timisoara, Calea Aradului No. 119, 300641 Timisoara, Romania

## Abstract

In recent years, nutraceuticals attracted a great amount of attention in the biomedical research due to their significant contribution as natural agents for prevention of various health issues. Ethanolic extracts from the ungerminated and germinated seeds of* Lupinus albus* L. and* Lupinus angustifolius* L. were analyzed for the content in isoflavones (genistein) and cinnamic acid derivatives. Additionally, the extracts were evaluated for antimicrobial, antiproliferative, and anti-inflammatory properties, using in vitro and in vivo tests. Germination proved to be a method of choice in increasing the amount of genistein and cinnamic acid derivatives in both* Lupinus albus *L. and* Lupinus angustifolius L.* seeds. Biological evaluation of all vegetal extracts revealed a weak therapeutic potential for both ungerminated and germinated seeds.

## 1. Introduction

Both traditional and complementary and alternative medicine of the 21 century have intensively shown the vital role of diet in maintaining human health [1, 2]. Nutraceuticals have been in the spotlight of research lately due to their significant contribution as natural agents for prevention of various health issues [3, 4].* Lupinus albus* L., commonly known as white lupin, and* Lupinus angustifolius* L., commonly known as the narrow-leafed lupin or blue lupin, members of the Fabaceae family, are two plant species described as nutraceuticals. The* Lupinus* genus comprises over 200 species. From the chemical point of view, the seeds contain approximately 36–52% proteins, 30–40% fibers, and 5–20% essential oils, with values depending on the environmental or genetic conditions [5, 6]. Other chemical constituents detected in* Lupinus* sp. were oleic and linoleic acids, isoflavonoids (genistein), carotenoids (zeaxanthin, thiamin, riboflavin, and niacin), and polysaccharides (galactan, cellulose, or hemicellulose). The amount of alkaloid content varies according to the species and the pedoclimatic conditions [6–8]. In terms of protein content,* Lupinus* species can be considered the strongest potential competitor of* Glycine max* L., soybean seeds; moreover,* Lupinus* species can be cultivated in Europe in contrast to soybean [9] which is most frequently found in Asia. Previous studies showed that the extract with high content of alkaloids obtained from* L. angustifolius* exhibited antimicrobial properties [10]. The group of Smart reported the allergy vaccine obtained from* L. angustifolius* which successfully suppressed experimental asthma [11]. The hypoglycemic effect of *γ*-conglutin-enriched lupin seeds extract was also depicted; *γ*-conglutin was described for its insulin-mimetic activity in mouse myoblasts [12, 13]. In a study developed on rats, it was demonstrated that the proteins from* L. albus* seeds were able to reduce cholesterol, consequently displaying cardiopreventive effects [14, 15]. Antioxidant effects of the seed extract were reported by Wang and Clements [16], while several* Lupinus* species displayed diuretic, anthelmintic, and emmenagogue activities [17].

The present study aimed (i) to detect the presence of cinnamic acid derivatives and genistein in the germinated and ungerminated seed extracts of two species of* Lupinus*,* L. albus* and* L. angustifolius*, and (ii) to screen the in vitro and in vivo effects of these extracts such as antiproliferative, antimicrobial, and anti-inflammatory activity.

## 2. Materials and Methods

### 2.1. Reagents

The reagents used in the present study were ethanol,methanol, and acetic acid of HPLC analytical grade from Merck (Germany), DMSO (dimethylsulfoxide), TPA (12-O-tetradecanoylphorbol-13-acetate), and acetone from Sigma-Aldrich. The standards for rosmarinic, ferulic, and caffeic acids and genistein were purchased from Sigma-Aldrich and for* p*-coumaric acid from Fluka. The media and the supplements for cell culture were achieved from Lonza Ltd., Basel, Switzerland.

### 2.2. Cell Lines

All cell lines were purchased from European Collection of Cell Cultures (ECCAC, Salisbury, UK) and were kept in standard conditions.

### 2.3. Animals

The animals used in the experiment were 8-week-old SKH-1 female mice. Mice were purchased from Charles River Laboratories (Budapest, Hungary). The work protocol followed all rules of National Institute of Animal Health (NIAH); animals were maintained during the experiment in standard conditions as follows: 12 h light-dark cycle, food and water ad libitum, temperature 22–24°C, and humidity around 55%.

### 2.4. Vegetal Extracts

*L. albus* and* L. angustifolius* seeds were grown in an experimental field of* Banat's University of Agricultural Sciences and Veterinary Medicine “King Michael I of Romania” from Timisoara* (21°13′E longitude, 45°45′N latitude) and harvested in September 2014. The ungerminated seeds were stored at room temperature in dry environment until analysis. For germination, hot water was poured over the seeds that were kept soaked for 3 days and then sown on moistened germination paper at 20°C, in the dark, for two weeks. Germinated and ungerminated seeds were then ground and submitted to extraction with ethanol 80% three times using an FALC LCD series ultrasonic bath for 10 min at 40 kHz and 28°C. The ratio between solvent and vegetal product was 1 : 3; the solvent was finally removed by using a rotary evaporator. The extracts obtained, ungerminated* L. albus* seeds extract (A), ungerminated* L. angustifolius* seeds extract (B), germinated* L. albus* seeds extract (C), and germinated* L. angustifolius* seeds extract (D), were further analyzed.

### 2.5. Cinnamic Acid (CA) Derivatives and Free Genistein (GY) Determination

The main CA derivatives (ferulic, caffeic, rosmarinic, and coumaric acids) were determined using LC-Shimadzu chromatograph equipped with degasser DGU-20AS, binary pump LC-20AD, SPD-10A UV detector, column thermostat CTO-20AC, and autosampler SIL 20-A. For the separation of compounds PERVAIL column 150 × 4.6 mm was used. The chromatographic conditions were as follows: (i) mobile phases A: methanol : acetic acid : water (90 : 2 : 8, v/v) and B: methanol : acetic acid : water (10 : 2 : 88, v/v), (ii) gradient program as follows: 0 min B 100%, at 10 min B 85%, at 15 min B 50%, at 20 min B 30%, and at 25 min B 80%, and (iii) the wavelengths 280 nm and 320 nm. The calibration curves for CA derivatives were established using dilutions in the range of 5–25 *μ*g/mL.

Genistein from extracts obtained was detected using LC-Shimadzu chromatograph equipped with SPD-10A UV detector and Adsorbosphere UHS C18 column. The chromatographic conditions were operated according to Hanganu et al.'s method [18]: (i) mobile phases A: acetic acid 0.1% (v/v) and B: methanol, (ii) linear gradient: until 2 min B 20%, at 10 min B 40%, and at 11.50 min B 45% and kept until 17 min at 45%, (iii) column temperature: 48°C, (iv) the flow rate 0.8 mL/min, and (v) the wavelengths—248 and 261 nm. The calibration curve of genistein was established using dilutions in the range of 5–100 *μ*g/mL.

### 2.6. In Vitro Antibacterial Activity

A volume of 50 mg/mL extracts obtained from germinated and ungerminated seeds of the two* Lupinus* species was screened for their antimicrobial activity against 5 bacterial strains,* Staphylococcus aureus* (ATCC 25923),* Pseudomonas aeruginosa* (ATCC 27853),* Escherichia coli* (ATCC 25922),* Klebsiella pneumoniae* (ATCC 700603), and* Staphylococcus epidermidis* (ATCC 14990), by means of the agar disk diffusion method. Chloramphenicol and fluconazole were used as reference.

### 2.7. Disk Diffusion Method

The antimicrobial activity of the extracts was determined by the disk diffusion method on Muller-Hinton agar using impregnated paper disks. Each bacterial strain was emulsified in sterile saline solution to obtain a concentration of 1.5 × 10^5^ CFU/mL. Muller-Hinton agar plates were inoculated with the respective microbial suspensions. The extracts were dissolved in 1 mL DMSO. Sterile paper disks (6 mm) were impregnated with 10 *μ*L of emulsified extract solution. These disks were placed on the surface of inoculated plates and incubated at 37°C for 24 h. The antimicrobial activity was assessed by measuring the clear inhibition area surrounding the tested extracts.

### 2.8. MTT Proliferation Assay

The antiproliferative effects of the prepared extracts were determined on a panel of human adherent cancer cell lines of gynecological origin. MCF7 and MDA-MB-231 cells were isolated from breast cancers, while A2780 and SiHa cells were isolated from ovarian and cervical malignancies, respectively. Cells were cultivated in minimal essential medium supplemented with 10% fetal bovine serum, 1% nonessential amino acids, and an antibiotic-antimycotic mixture (Lonza Ltd., Basel, Switzerland). Cancer cells were seeded in a 96-well microplate at the density of 5000/well and allowed to adhere overnight and 200 *μ*L new medium containing the tested extract was added. After incubation for 72 h at 37°C in humidified air containing 5% CO_2_, the percentage of living cells was assessed after addition of 20 *μ*L MTT solution. The medium was removed and the precipitated formazan crystals were dissolved in 100 *μ*L DMSO during a 60 min period of shaking at 37°C. The absorbance values were read at 545 nm, using a microplate reader; wells with untreated cells were utilized as control and inhibition % values were calculated. All in vitro experiments were carried out on two microplates with at least five parallel wells. Cisplatin was used as positive control. Stock solutions of the tested substances (50 mM) were prepared in DMSO.

### 2.9. Experimental Design of the Study

The animal model of ear inflammation was performed on SKH-1 hairless mice. The protocol applied was as the one described in the literature [19] with several modifications: the ear edema was induced by topical application of TPA 2 *μ*g/ear dissolved in 20 *μ*L acetone. The volume of TPA solution was applied on both the inner and the outer surfaces of the mouse ear. The mice were divided in the following groups: control group, Group A (mice topically treated with acetone), Group B, mice topically treated with TPA solution, Group C, mice topically treated with TPA solution followed after 30 minutes by the application of 100 *μ*L ungerminated* L. albus* seeds extract (50 mg/mL), Group D, mice topically treated with TPA solution followed after 30 minutes by the application of 100 *μ*L germinated* L. albus* seeds extract (50 mg/mL), Group E, mice topically treated with TPA solution followed after 30 minutes by the application of 100 *μ*L of ungerminated* L. angustifolius* seeds extract (50 mg/mL), and Group F, mice topically treated with TPA solution followed after 30 minutes by the application of 100 *μ*L of germinated* L. angustifolius* seeds extract (50 mg/mL). At 4 h after application of the test extracts, the mice were sacrificed under anesthesia by cervical dislocation and 6 mm^2^ diameter ear punch biopsies were collected and histopathologically analyzed.

### 2.10. Histopathological Analysis

Samples of mouse ear were fixed in 10% v/v buffered formalin solution and then embedded in paraffin. Six-micrometer-thick serial slides were sectioned and stained with the conventional hematoxylin-eosin dye (H&E). Image acquisition and analysis were performed using a Nikon Eclipse E600 microscope. Inflammation was quantified as 1, mild; 2, moderate; and 3, severe.

### 2.11. Statistics

The Prism software package (GraphPad Prism 4.03 for Windows) was used for data presentation. The experiment was repeated three times and results were presented as mean ± SD. Paired Student's *t*-tests and one-way or two-way ANOVA were applied to evaluate statistical significance (^*∗*^*p* < 0.05; ^*∗∗*^*p* < 0.01; and ^*∗∗∗*^*p* < 0.001).

## 3. Results and Discussions

**Figure 1:**
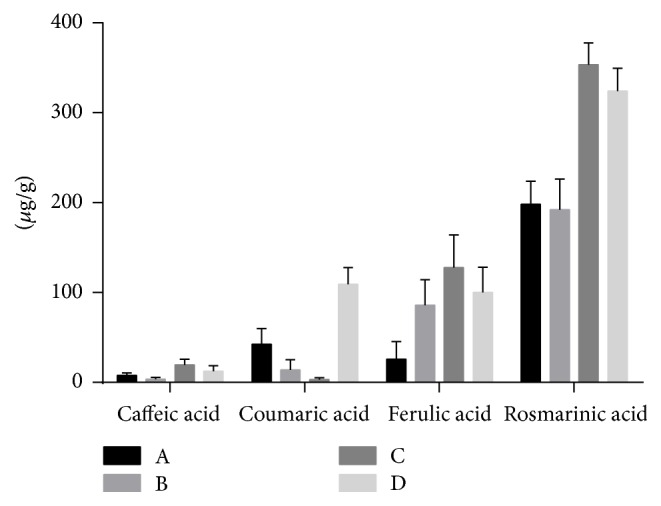
Cinnamic acid (CA) derivatives determination of the selected extracts expressed as *μ*g/g (two-way ANOVA, Interaction, *p* = 0.0022, Row Factor, *p* < 0.0001, and Column Factor, *p* = 0.0002).

One of the objectives of the present study consisted in detecting the presence of cinnamic acid derivatives and genistein in germinated and ungerminated seed extracts of* L. albus* and* L. angustifolius*. According to our results, it was shown that the caffeic acid content was increased in germinated extracts as compared to ungerminated ones as follows: 1.304 ± 0.421 *μ*g/g (A, ungerminated* L. albus* seeds extract) versus 3.389 ± 1.053 *μ*g/g (C, germinated* L. albus *seeds extract) and 0.531 ± 0.331 *μ*g/g (B, ungerminated* L. angustifolius* seeds extract) versus 2.114 ± 1.003 *μ*g/g (D, germinated* L. angustifolius* seeds extract). Regarding the content of the other three cinnamic acid derivatives (coumaric, ferulic, and rosmarinic acids), an increase in the germinated extracts of* L. angustifolius* was determined, with the highest value being obtained for rosmarinic acid (coumaric acid < ferulic acid < rosmarinic acid; Figure 1). The germinated extracts of* L. albus* induced an increase of ferulic and rosmarinic acids content, whereas the coumaric acid content was reduced (Figure 1). In the present study, caffeic and coumaric acids were found in low concentrations in both germinated and ungerminated seeds as compared to the ferulic and rosmarinic acids. Similar results were reported by Siger et al. in a study using ungerminated seeds; according to these authors, the content of caffeic acid was 0.58 *μ*g/g in* L. albus* seeds and 0.84 *μ*g/g in* L. angustifolius* seeds [20]. The presence of cinnamic acid derivatives in germinated and ungerminated seeds of* Lupinus* sp. was also reported by other research groups [21, 22]. Luthria and Pastor-Coralles described in 2005 the presence of ferulic and coumaric acids in several varieties of* Phaseolus vulgaris* Linnaeus beans in the range of 106–229 *μ*g/g for ferulic acid and 17–124 *μ*g/g for coumaric acid [23]. Siger et al. showed that germination changes the quantitative and qualitative polyphenolic composition of lupin seeds, significantly increasing the flavonoids amount [20].

**Figure 2:**
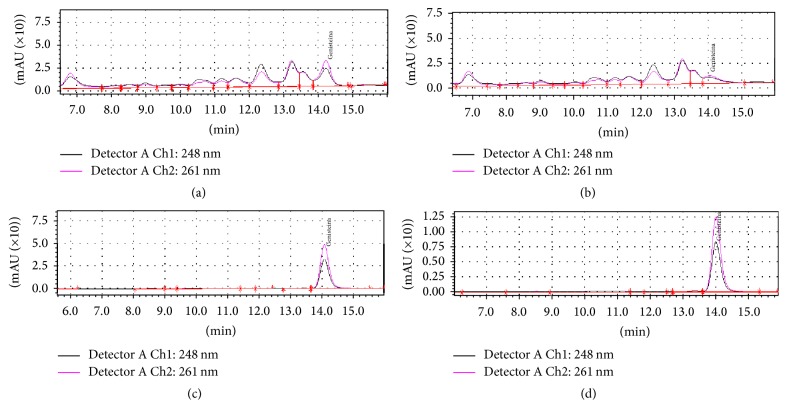
HPLC chromatogram for GY determination: (a) extract from ungerminated seed of* Lupinus albus*; (b)* Lupinus angustifolius*; (c) germinated seed of* Lupinus albus*; (d)* Lupinus angustifolius*. GY: genistein. Data collected at 261 nm (pink line) and 248 nm (black line).

HPLC analysis was performed in order to detect the expression of genistein, the most important isoflavone present in both germinated and ungerminated seeds of the two* Lupinus* sp. The HPLC chromatograms of germinated and ungerminated lupin seeds are presented in Figure 2. The validation parameters (the regression equations, retention times, limits of detection, and limits of quantification) for CA derivatives and GY were presented in Table 1. The limit of detection (LOD) represents the amount of compounds that could be detected with a signal-to-noise ratio (S/N ≥ 3), while the limit of quantification (LOQ) represents the lowest concentration for which S/N ≥ 5. Intraday and interday accuracy and precision values of the method were detected. In determining the intraday accuracy and precision, six samples were injected within the same day. This procedure was repeated once a day for 3 consecutive days to evaluate the interday precision. The intraday coefficient of variation (RSD %) ranged from 2.50 to 14.5% and the accuracy from 94.4 to 98.0%, while the interday RDS (%) varied between 6.54 and 16.1% and the accuracy from 93.5 to 97.4%. The results indicated that the assay was reproducible and accurate.

**Figure 3:**
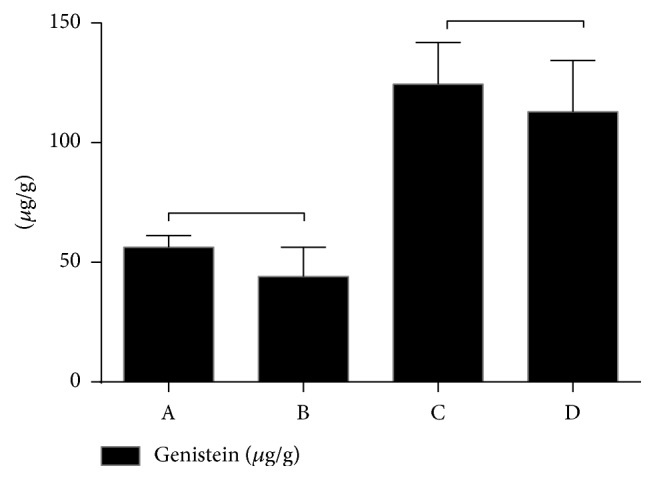
Genistein amount (*μ*g/g) in the analyzed samples (paired Student's *t*-test, *p* < 0.029, respectively, *p* < 0.036).

The results presented in Figure 3 showed that* L. albus* seeds extract (A) had a slightly higher genistein concentration as compared to* L. angustifolius* seeds extract (B), namely, 9.395 ± 0.813 *μ*g/g versus 7.335 ± 2.043 *μ*g/g, values that indicate a relatively low amount of genistein. The two weeks of germination significantly increased genistein concentration as follows: 20.74 ± 2.899 *μ*g/g genistein in germinated* L. albus* seeds extract (C) and 18.83 ± 3.549 *μ*g/g genistein in germinated* L. angustifolius* seeds extract (D). Genistein content in several cultivars of* L. albus* stems varies in a large range: 0.2–27.44 ppm [24], 48–84 ppm [25], and 116.1 ppm in seedlings [26], while the reported values in seeds are lower (1.5 ppm) [27]. Germination induces the increase by five times of the amount of isoflavones in soybeans after 1 week of germination, mechanism that can be explained through the activation of b-glucosidase enzymes in the soaking step prior to germination or by the germination metabolism [28].

The selected extracts were assessed for their antimicrobial potential against five bacterial strains:* S. aureus* (ATCC 25923),* P. aeruginosa* (ATCC 27853),* E. coli* (ATCC 25922),* K. pneumoniae* (ATCC 700603), and* S. epidermidis* (ATCC 14990). Chloramphenicol 30 *μ*g and fluconazole 25 *μ*g were used as references. Samples showed no antimicrobial activity on the tested strains except for sample D which created a 15 mm zone of inhibition on* K. pneumonia* strain. The results were presented in Table 2. The extracts of both germinated and ungerminated seeds of the two* Lupinus* sp. show poor antimicrobial activity on the selected bacterial strains. Previous papers reported the antibacterial and antifungal activity of the alkaloid extract of* L. angustifolius* seeds. The extract exhibited significant activity on* B. subtilis, S. aureus*, and* P. aeruginosa* at MICs of 62.5 *μ*g/mL [10]. Lampart-Szczapa et al. showed that extracts obtained from three species of* Lupin* sp. (*albus*,* luteus*, and* angustifolius*) seeds cotyledons did not exhibit any antibacterial activity against* E. coli* and* B. subtilis*, whereas extracts obtained from testas induced inhibition zones between 14 and 27 mm. They also reported the direct correlation between the antibacterial activity and the amount of free phenolic acids [9]. Current data correlated with poor literature reports in terms of the antimicrobial activity of various* Lupinus *sp. seeds allowed us to conclude that extracts from the seeds of these species are not the first choice as antimicrobial agents. On the other hand, extracts from* L. arboreus *leaves exhibited a broad spectrum of activity against certain bacterial strains [29]. Also, the root extract of Sudanese* L. termis* showed moderate antimicrobial activity [30].

The four extracts obtained in the present study were analyzed for their antiproliferative capacity against four cancer cell lines: MCF7, MDA-MB-231 (breast cancers), A2780 (ovarian cancer), and SiHa (cervical cancer). Our results showed a poor antiproliferative activity as depicted in Table 3. None of the selected extracts was active against the A2780 ovarian cancer cell line. For the breast cancer cell lines MCF7 and MDA-MB-231, the results showed an antiproliferative activity only at the highest concentration used in the case of ungerminated* L. angustifolius* seeds extract (B). A similar effect was found at the same concentration for the germinated* L. angustifolius* seeds extract (D). According to our data, germination does not seem to induce a significant effect on the antiproliferative capacity against the tumor cell lines tested in the study. SiHa cervical cancer cell line seems to be more sensitive to the tested extracts; the antiproliferative activities elicited by the highest concentration of extracts (C) and (D) were around 30%. Previous studies in this field reported that a volume of 200 *μ*g/mL ethanolic extracts from the root and shoot of* L. angustifolius* displayed antiproliferative activity against MCF-7 and BT20 breast cancers cell lines [31]; additionally, genistein-8-C-glucoside from* L. luteus* induced DNA damage and reduced cell viability of the mouse embryonic fibroblast, NIH 3T3 cell line, when used in concentrations >20 *μ*M [32].

**Figure 4:**
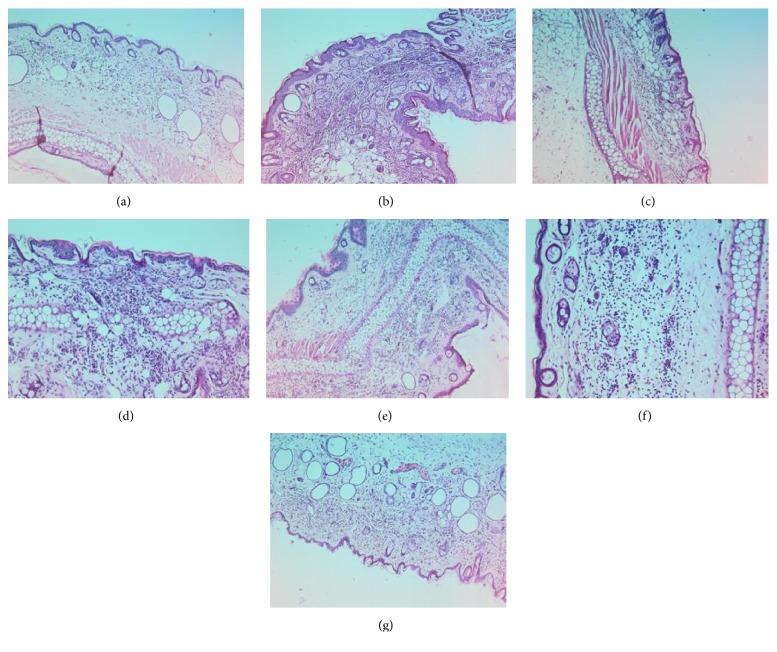
Ear sections corresponding to groups (a)–(g) were stained with H&E and analyzed for inflammatory response. Magnification 20x; scale bar: 150 *μ*m.

Histological analysis of the ears revealed the presence of an infiltrate of mixed inflammatory cells, with the polymorphonuclear leukocytes being more abundant and only few lymphocytes. One can notice that ear samples collected from groups A, C, F, and G showed moderate inflammation, while groups B, D, and E disclosed severe inflammatory response, with abundant cell influx (Figure 4).

**Figure 5:**
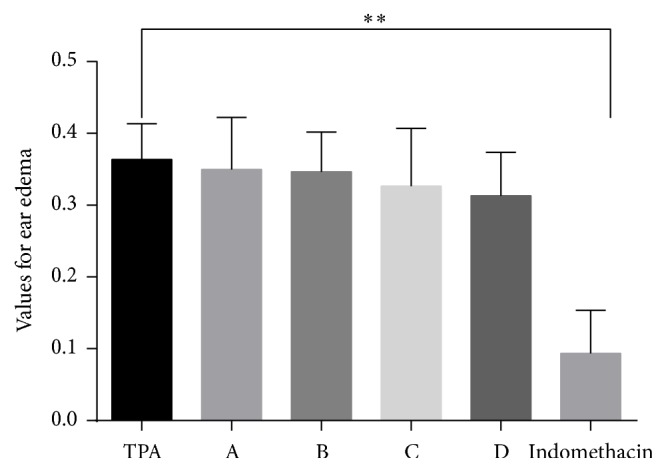
Ear edema values for extracts A, B, C, and D using TPA as blank and indomethacin 1% as reference for the anti-inflammatory activity (one-way ANOVA, *p* = 0.0019, *∗∗*).

The values recorded for ear edema in the tested groups are shown in Figure 5. No anti-inflammatory potential was noticed for the ungerminated seeds extracts of the two* Lupinus* sp. Germinated seeds of both species presented mild anti-inflammatory activity. These results confirmed the H&E findings. Studies on animal models showed the mild anti-inflammatory activity provided by the extracts from germinated seeds of the two* Lupinus* sp.; recent in vitro studies have also reported the anti-inflammatory activity of protein hydrolysates from* L. angustifolius* in THP-1-derived macrophages [33]. In another study focusing on the assessment of topical applications of curcuminoids and flavonoids on male albino rats, the group of Hamzah showed that the decrease of edema percentage induced by ethanol fraction lupines was 18%, while chloroform fraction lupines led to 11.3% edema inhibition [34]. Additionally, aqueous* L. mutabilis* sweet extract was described to produce gastric anti-inflammatory and antisecretory activities in rats [35]. These results confirmed and completed the data existing in the literature regarding the biological characterization of the compounds and in vitro and in vivo evaluation of germinated and ungerminated seeds extract from the two* Lupinus* species* L. albus* and* L. angustifolius*.

## 4. Conclusion

The study showed that germination is an excellent natural method that can be employed for the augmentation of some active plant secondary metabolites content as isoflavones and cinnamic acid derivatives in nutraceuticals like* L. albus* and* L. angustifolius* seeds. Biological evaluation of the antimicrobial, antiproliferative, and anti-inflammatory activity of all vegetal extracts revealed a weak therapeutic potential for both ungerminated and germinated seeds. As described in the present study, germinated seed of different* Lupinus* sp. can represent a valuable source for the intake of genistein and cinnamic acid derivatives and directly correlated their beneficial effects on the human body.

## Figures and Tables

**Table 1:** Validation parameters for cinnamic acid (CA) derivatives and free genistein (GY).

Compounds	Retention time (*r*_*T*_)	Regression equations	Regression coefficient (*R*^2^)	LOD (*µ*g/mL)	LOQ (*µ*g/mL)
Caffeic acid	10.4	*y* = 2.36*x* − 2.34	0.998^†^	^†^0.5	0.7
Coumaric acid	15.4	*y* = 2.00*x* − 2.65	0.996	0.5	0.7
Ferulic acid	16.1	*y* = 2.96*x* − 5.28	0.989	0.5	0.7
Rosmarinic acid	17.2	*y* = 4.22*x* − 2.02	0.990	0.4	0.6
Genistein	14.0	*y* = 1.77*x* − 3.95	0.986	0.4	0.6

**Table 2:** Antimicrobial activity of the selected extracts expressed as zone of inhibition (mm).

Reference strain	Chloramphenicol 30 *µ*g	Fluconazole 25 *µ*g	A	B	C	D
Zone of inhibition (mm)						
*S. aureus* ATCC 25923	30	0	0	0	0	0
*P. aeruginosa* ATCC 7853	0	0	0	0	0	0
*E. coli* ATCC 25922	30	15	0	0	0	0
*K. pneumoniae* ATCC 700603	16	0	0	0	0	15
*S. epidermidis* ATCC 14990	30	0	0	0	0	0

**Table 3:** Antiproliferative activity of the selected extracts expressed as inhibition % against the mentioned cell lines. Extracts eliciting less than 10% inhibition are considered ineffective and the exact values were not presented for clarity purposes.

Extract	Final concentration	Inhibition (%) ± SEM			
		A2780	MCF7	MDA-MB-231	SiHa
A	15 *µ*g/mL	—	—	—	—
	50 *µ*g/mL	—	—	—	—
	150 *µ*g/mL	—	—	—	11.42 ± 1.55

B	15 *µ*g/mL	—	—	—	—
	50 *µ*g/mL	—	—	—	—
	150 *µ*g/mL	—	10.54 ± 2.46	11.40 ± 1.99	18.23 ± 3.23

C	15 *µ*g/mL	—	—	—	—
	50 *µ*g/mL	—	—	—	11.65 ± 3.07
	150 *µ*g/mL	—	—	—	27.09 ± 0.85

D	15 *µ*g/mL	—	—	—	—
	50 *µ*g/mL	—	—	—	11.26 ± 2.26
	150 *µ*g/mL	—	16.83 ± 3.55	10.83 ± 1.10	32.53 ± 1.49
